# Effects of nicotine on corneal wound healing following acute alkali burn

**DOI:** 10.1371/journal.pone.0179982

**Published:** 2017-06-23

**Authors:** Jong Won Kim, Chae Woong Lim, Bumseok Kim

**Affiliations:** Biosafety Research Institute and Laboratory of Pathology (BK21 Plus Program), College of Veterinary Medicine, Chonbuk National University, Iksan, Republic of Korea; Heart Research Institute, AUSTRALIA

## Abstract

Epidemiological studies have indicated that smoking is a pivotal risk factor for the progression of several chronic diseases. Nicotine, the addictive component of cigarettes, has powerful pathophysiological properties in the body. Although the effects of cigarette smoking on corneal re-epithelialization have been studied, the effects of nicotine on corneal wound healing-related neovascularization and fibrosis have not been fully demonstrated. The aim of this study was to evaluate the effects of chronic administration of nicotine on corneal wound healing following acute insult induced by an alkali burn. BALB/C female mice randomly received either vehicle (2% saccharin) or nicotine (100 or 200 μg/ml in 2% saccharin) in drinking water *ad libitum*. After 1 week, animals were re-randomized and the experimental group was subjected to a corneal alkali burn, and then nicotine was administered until day 14 after the alkali burn. A corneal alkali burn model was generated by placing a piece of 2 mm-diameter filter paper soaked in 1N NaOH on the right eye. Histopathological analysis and the expression level of the pro-angiogenic genes vascular endothelial growth factor (VEGF) and matrix metalloproteinase-9 (MMP9) revealed that chronic nicotine administration enhanced alkali burn-induced corneal neovascularization. Furthermore, the mRNA expression of the pro-fibrogenic factors α-smooth muscle actin (αSMA), transforming growth factor-β (TGF-β), and collagen α1 (Col1) was enhanced in the high-concentration nicotine-treated group compared with the vehicle group after corneal injury. Immunohistochemical analysis also showed that the αSMA-positive area was increased in chronic nicotine-treated mice after corneal alkali burn. An *in vitro* assay found that expression of the α3, α7, and β1 nicotinic acetylcholine receptor (nAChR) subunits was significantly increased by chemical injury in human corneal fibroblast cells. Moreover, alkali-induced fibrogenic gene expression and proliferation of fibroblast cells were further increased by treatment with nicotine and cotinine. The proliferation of such cells induced by treatment of nicotine and cotinine was reduced by inhibition of the PI3K and PKC pathways using specific inhibitors. In conclusion, chronic administration of nicotine accelerated the angiogenic and fibrogenic healing processes in alkali-burned corneal tissue.

## Introduction

Cigarette smoking, the most common form of tobacco use, is responsible for hundreds of thousands of premature deaths and chronic diseases annually [1]. Many epidemiological studies have shown that cigarette smoke (CS) is positively correlated with increased human mortality via various chronic diseases, including renal, cardiovascular, pulmonary, and hepatic diseases [2–5], as well as several forms of cancer [6]. CS is made up of a variety of constituents and exerts many pathophysiological roles. Of these, nicotine, the addictive component of cigarettes, binds to its receptors, which have several subtypes (α1–10, β1–4, δ, γ, and ε), and thereby exerts a direct action on neuronal and non-neuronal cells [7]. Nicotine has many biological effects, such as inflammation [8] or anti-inflammation [9], cell proliferation, apoptosis, angiogenesis [10], and fibrosis [7, 11, 12].

The wound healing response induced by tissue damage is a complex and dynamic process involving various extracellular matrix proteins, growth factors, and cytokines. The three phases of wound healing are defined as inflammation, tissue formation (proliferation), and maturation (tissue remodeling), which temporally overlap. Inflammation is characterized by recruitment of neutrophils and macrophages to an injured site in response to chemokines. The main and leading events that happen during the second phase of wound healing, which is known as tissue formation or the proliferative phase, include epithermal restoration, formation of new blood vessels, fibroblast proliferation, and production of extracellular matrix (ECM). Tissue remodeling is the last stage of wound healing and involves vascular regression and a progressive remodeling of granulation tissue [13–15]. However, excess inflammation and tissue fibrosis/scarring during the wound healing response disrupt the normal function of tissues and organs [16].

Corneal tissue is characterized by an absence of both lymphatic and blood vessels under normal conditions, which allows for optical clarity and visual acuity [17]. Infections or trauma induced by chemical/surgical injury to the cornea can cause neovascularization (NV) and fibrosis/scarring, resulting in loss of corneal transparency with consequent permanent visual impairment. The wound healing response of alkali-burned cornea is characterized in part by infiltration of macrophages/monocytes and neutrophils into the corneal stroma from the limbus and changes in cellular phenotypes. However, inhibition of CXCR2-mediated neutrophil infiltration failed to attenuate alkali injury-induced corneal NV, indicating that corneal NV could occur independently of neutrophil accumulation [18]. Furthermore, keratocyte activation, myofibroblast formation, and subsequent neovascularization and tissue fibrosis are all involved in the wound healing response or scar formation in an alkali-burned cornea [19, 20].

While normal corneal keratocytes are quiescent and contribute to the maintenance of corneal transparency, disruption of the integrity of the cornea caused by alkali injury induces differentiation of quiescent cells into fibroblasts and/or myofibroblasts. Corneal myofibroblasts have a different morphology than do keratocytes and express α-smooth muscle actin (α-SMA) [21]. Among the numerous cytokines and growth factors that affect the wound healing response, transforming growth factor-β (TGF-β) plays a critical role in regeneration of damaged tissue. In particular, TGF-β1 is a potent stimulator of type I collagen (Col1) in fibroblasts, and, concomitantly, it also inhibits the expression of several matrix metalloproteinases (MMPs), which further prompt the accumulation of collagen fibers and formation of fibrotic tissues [22]. Also, TGF-β1 is a potent inducer of the differentiation of fibroblasts to myofibroblasts. These differentiated cells play a pivotal role in wound repair by secreting ECM proteins, including several types of collagen. However, the wound healing response in corneal tissue is closely associated with corneal haze, scar formation, and surface irregularities after corneal surgery, infection, and injury [23].

A recent study indicated that epithelial healing is delayed in smokers as compared with nonsmokers with corneal abrasions and keratitis. Neurotrophic corneas and fungal infections also prolonged the time necessary for healing in smokers [24]. In addition, it is known that second-hand CS, a form of CS inhaled by passive smokers, delays corneal re-epithelialization and healing in mice by stimulating inflammation and degradation of the ECM [25]. Furthermore, activation of nicotinic acetylcholine receptors (nAChRs) by nicotine promotes choroidal NV and may contribute to the increased incidence of choroidal neovessel formation seen in smokers with age-related macular degeneration (AMD) [26]. However, little is known regarding the effects of nicotine on corneal NV and fibrosis induced by chemical burns. Therefore, our study aimed to determine such effects in an alkali corneal injury model.

## Materials and methods

### Animals

Eight-week-old female BALB/c mice were purchased from Samtako (South Korea). Mice were maintained in a standard condition (24 ± 2°C, 50 ± 5% humidity), pathogen-free environment with a 12-hour light/dark cycle. Experimental and animal management procedures were approved by the Institutional Animal Care and Use Committee of Chonbuk National University. The animal facility of Chonbuk National University is fully accredited by the National Association of Laboratory Animal Care.

### Experimental protocol and alkali-induced corneal injury model

In the nicotine-treated groups, mice (n = 6–7 mice/group) received (-)-nicotine (Sigma-Aldrich, St.Louis, MO, USA) in their drinking water (100 μg/ml or 200 μg/ml in a 2% saccharin (Sigma-Aldrich). Two percent saccharin solution alone was administered to the control group. Administration of nicotine at a concentration of 100 μg/ml provides a serum nicotine level (216.5 ng/mL; 95% CI, 189.5 to 236.6) equivalent to that of a one pack a day smoker [27]. After 1 week, mice were anesthetized with an i.m. injection of Zoletil at a dose of 0.2 ml/Kg body weight. Following topical application of a drop of 0.5% proparacaine hydrochloride (Sigma-Aldrich) to the corneal surface for local analgesia, a piece of Whatman #3 filter paper (2 mm in diameter) soaked in 1N NaOH was placed on the center of the left cornea for 40 seconds. The ocular surface was then rinsed with 25 ml of PBS. After the alkali burn injury, mice were euthanized by cervical dislocation following anesthesia, and the corneas were removed at the indicated time intervals (4, 7, and 14 days). The corneas were stored at -80°C and used for RNA extraction. In another series of experiments, mice were sacrificed at the abovementioned times after alkali treatment and eyes were entirely removed from each animal. These eyes were fixed in 10% phosphate-buffered formalin for histopathological analysis.

### Microscopic examination

Eyes were examined with a stereomicroscope (SZ61, Olympus Corp., Tokyo, Japan) on days 7 and 14 after alkali injury. The length of corneal new blood vessels was measured by using digital imaging software (analySIS TS, Olympus Corp., Tokyo, Japan). Microscopic assessment was done by observers without prior knowledge of the procedures.

### Quantitative real time polymerase chain reaction (qRT-PCR)

Total RNA was isolated from tissues using the Easy-Spin Total RNA extraction kit (GeneAll, Seoul, Korea). Following incubation with RNase-free DNase I (Promega, Madison, WI, USA), reverse transcription was performed using a random primer and MultiScribe^™^ MuLV reverse transcriptase (Applied Biosystems, Foster City, CA, USA) according to the manufacturer’s instructions. cDNA was subjected to qRT-PCR on a CFX96^™^ Real-Time PCR Detection System (Bio-Rad Laboratories, CA, USA) using SYBR Green I as a double-stranded DNA-specific binding dye. After the reaction was complete, specificity was verified by melting curve analysis. Quantification was performed by normalizing the Ct values of each sample to murine or human glyceraldehyde-3-phosphate dehydrogenase (GAPDH or hGAPDH). The sequences of the PCR primers used are given in Tables 1 and 2.

**Table 1:** Primer sequence of genes used for qRT-PCR.

Gene	Forward	Reverse
VEGF	5’-CTGGATATGTTTGACTGCTGTGGA-3’	5’-GTTTCTGGAAGTGAGCCAATGTG-3’
MMP9	5’-GCCCTGGAACTCACACGACA-3’	5’-TCTGAGCGATGCCATCAAAGAC-3’
αSMA	5’-TCAGGGAGTAATGGTTGGAA-3’	5’-CAGTTGGTGATGATGCCGTG-3’
Col1	5’-ACAGGCGAAACCGGTGACAG-3’	5’-GCCAGGAGAACCAGCAGAGC-3’
TGF-β	5’-TGAACCAAGGAGACGGAATACAGG-3’	5’-GCCATGAGGAGCAGGAAGGG-3’
α3	5’-ACCCTTGATGGCCAGAGATGA-3’	5’-TCCAAGCAGTGATCGGATAAAGTG-3’
α7	5’-AACCATGCGCCGTAGGACA-3’	5’-CTCAGCCACAAGCAGCATGAA-3’
β1	5’-TTCATCCGGAAGCCTCCAA-3’	5’-TGAAGCTGTCGGGCCATGTA-3’
GAPDH	5’-ACGGCAAATTCAACGGCACAG-3’	5’-AGACTCCACGACATACTCAGCAC-3’

**Table 2:** Primer sequence of human nAChRs used for qRT-PCR.

Gene	Forward	Reverse
α1	5’-GCAGAGACCATGAAGTCAGACCA-3’	5’-CGACCTGCAAACACGGCTA-3’
α2	5’-GATTTGGAGATGAGCCCAAAGTG-3’	5’-ACCCTGCAAACTCGGACAGAC-3’
α3	5’-CTTGCTGCTCACCTGGAAAT-3’	5’-TATGGTGGGGCAGAGTTCAT-3’
α4	5’-GTGGATGAGAAGAACCAGATGATG-3’	5’-CAGCGCAGCTTGTAGTCGTG-3’
α5	5’-AGATGGAACCCTGATGACTATGGT-3’	5’-AAACGTCCATCTGCATTATCAAAC-3’
α6	5’-GCCTGAAGTTGAAGATGTGA-3’	5’-ATACTCTGTCCACCACCAT-3’
α7	5’-GCTGCTCGTGGCTGAGATC-3’	5’-TGGCGAAGTACTGGGCTATCA-3’
α9	5’-TTTCAGCTAATGGTGGCAGAAATC-3’	5’-ATCAGGGCCATCGTGGCTA-3’
α10	5’-CGCTCACCGTCTTCCAGTT-3’	5’-TGCAGGTTCATGATAAGGATGGT-3’
β1	5’-CTCGACAGCTGCAGGAACA-3’	5’-CAACGCTGGTGAAGATGATG-3’
β2	5’-CCTTTGGGAACCATCACAATAA-3’	5’-TCCCACCACATGGTAGCAC-3’
β3	5’-TCCTCAGACATTTGTTCCAAGGTTA-3’	5’-GCCACACATTGGTTGTCATCAG-3’
β4	5’-GATGTGCAGGAGGCATTAGAAGGT-3’	5’-GAAGCTGCATGGGTCTGGAAG-3’
γ	5’-CTGTGTGGAAGCCTGCAACC-3’	5’-CCACCAGGAACCACTCCTCATT-3’
δ	5’-GAGAGAGGAGCCACAGTC-3’	5’-GCCATTGAGCATTGAGGAG-3’
ε	5’-CATCAGCCTCAAGGTCAC-3’	5’-CGGTAATCCTGCCAATCG-3’
^a^hGAPDH	5’-GCACCGTCAAGGCTGAGAAC-3’	5’-TGGTGAAGACGCCAGTGGA-3’

^a^, human GAPDH

### Immunohistochemical analysis

To detect αSMA expression in injured corneas, immunohistochemical staining was conducted. In brief, corneal tissue sections were incubated with rabbit anti-αSMA antibody (Abcam, Cambridge, UK) overnight at 4°C. Negative control slides were incubated with non-immune immunoglobulin under the same conditions. The sections were further incubated with biotin-conjugated rat or rabbit Ig secondary antibody. The immune complexes were detected using the DAB Substrate Kit from Vector Laboratories according to the manufacturer’s instructions. Finally, the tissues were counterstained with hematoxylin and mounted. The number of positive cells per square millimeter was counted in five randomly selected fields of the immunohistochemically stained slides at 200-fold magnification. Images were analyzed using a light microscope (BX-51, Olympus Corp., Tokyo, Japan) and digital image software (analySIS TS, Olympus Corp., Tokyo, Japan).

### Western blot assay

Corneal tissues and cells were directly lysed for 30 minutes on ice with an extraction buffer (T-PER and RIPA, Thermo Fisher Scientific Inc., Waltham, IL, USA). After centrifugation at 13,000 g for 15 minutes at 4°C, the protein concentration in the supernatant was measured using the Pierce BCA Protein Assay kit (Thermo Fisher Scientific Inc., Waltham, IL, USA) according to the manufacturer’s protocol. Protein was loaded onto sodium dodecyl sulfate-polyacrylamide gel electrophoresis (SDS-PAGE) gels, transferred to polyvinylidene difluoride (PVDF) membranes, and subsequently blocked with 5% nonfat dried milk in Tris-buffered saline (20 mM Tris, 150 mM NaCl, pH 7.4) with 0.05% Tween-20 for 1 hour at RT. Primary antibodies were diluted 1:1000 in blocking solution and incubated overnight at 4°C. The following antibodies were used: rabbit anti-αSMA antibody (Abcam, Cambridge, UK) and anti-β-actin (Santa Cruz Biotechnology Inc., Dallas, TX, USA). To detect antigen-antibody complexes, peroxidase-conjugated secondary antibodies (Santa Cruz Biotechnology Inc., Dallas, TX, USA) were diluted 1:2000 in blocking solution and incubated for 1 hour at RT. The protein bands were visualized with an enhanced chemiluminescence (ECL) detection system using ImageQuant^™^ LAS 500 (GE healthcare Life Science, Pittsburgh, PA, USA). Protein expression levels were quantified with ImageQuant^™^ TL software.

### Cell culture

The human corneal fibroblast cell line was kindly provided by Prof. Eung Kweon Kim (University of Yonsei, Seoul, South Korea). Cells were cultured in Dulbecco’s modified Eagle medium (DMEM; Thermo Fisher Scientific Inc., Waltham, IL, USA) with high glucose, 10% FBS, 100 IU/ml penicillin, and 100 μg/ml streptomycin at 37°C in a 5% CO_2_ humidified incubator. The cells were cultured until 80% confluent and the medium was changed every 2 days. The cells were routinely passaged by trypsinization using 0.05% trypsin. Cells were seeded in wells of a 12-well plate (2×10^5^ cells/ml) with 1 ml medium per well, and then treated with 0.01 N NaOH with or without the indicated concentrations of nicotine or cotinine (Sigma-Aldrich, St. Louis, MO, USA) in order to mimic an alkali burn corneal injury, as previously described [28]. The plates were incubated for 24 hours in a 37°C humidified incubator containing 5% CO_2_. After being cultured for a defined time, the samples were used for other characterizations.

### Cell proliferation assay

Cells were seeded in wells of a 96-well plate (5×10^4^ cells/ml) with 100 μl medium per well, and then allowed to adhere and grow for 24 h, followed by treatment with the indicated concentrations of nicotine or cotinine and other materials. Ro-32-0432 (RO; Santa Cruz Biotechnology Inc., Dallas, TX, USA) and Wortmannin (WO; Santa Cruz Biotechnology Inc., Dallas, TX, USA) were used to inhibit protein kinase C (PKC) and phosphoinositide 3-kinase (PI3K), respectively. After 24 hours of incubation, cell proliferation was evaluated using a CCK-8 (Dojindo Molecular Technologies Inc. Rockville, MD, USA) according to the manufacturer’s instructions. The absorbance of samples was read with a spectrophotometer (EMax, Molecular Devices, Sunnyvale, CA, USA).

### Statistical analysis

All data are expressed as means ± standard errors. Differences between multiple groups were compared using one-way analysis of variance (ANOVA) with SAS version 9.1 (SAS Institute Inc., Cary, NC, USA); individual comparisons were obtained by Duncan's Multiple Range Test (DMRT). Differences between two groups were compared using a two-tailed Student’s t-test. A value of p<0.05 was considered statistically significant.

## Results

### Nicotine administration promotes new blood vessel formation in corneas after an alkali burn injury

**Fig 1:**
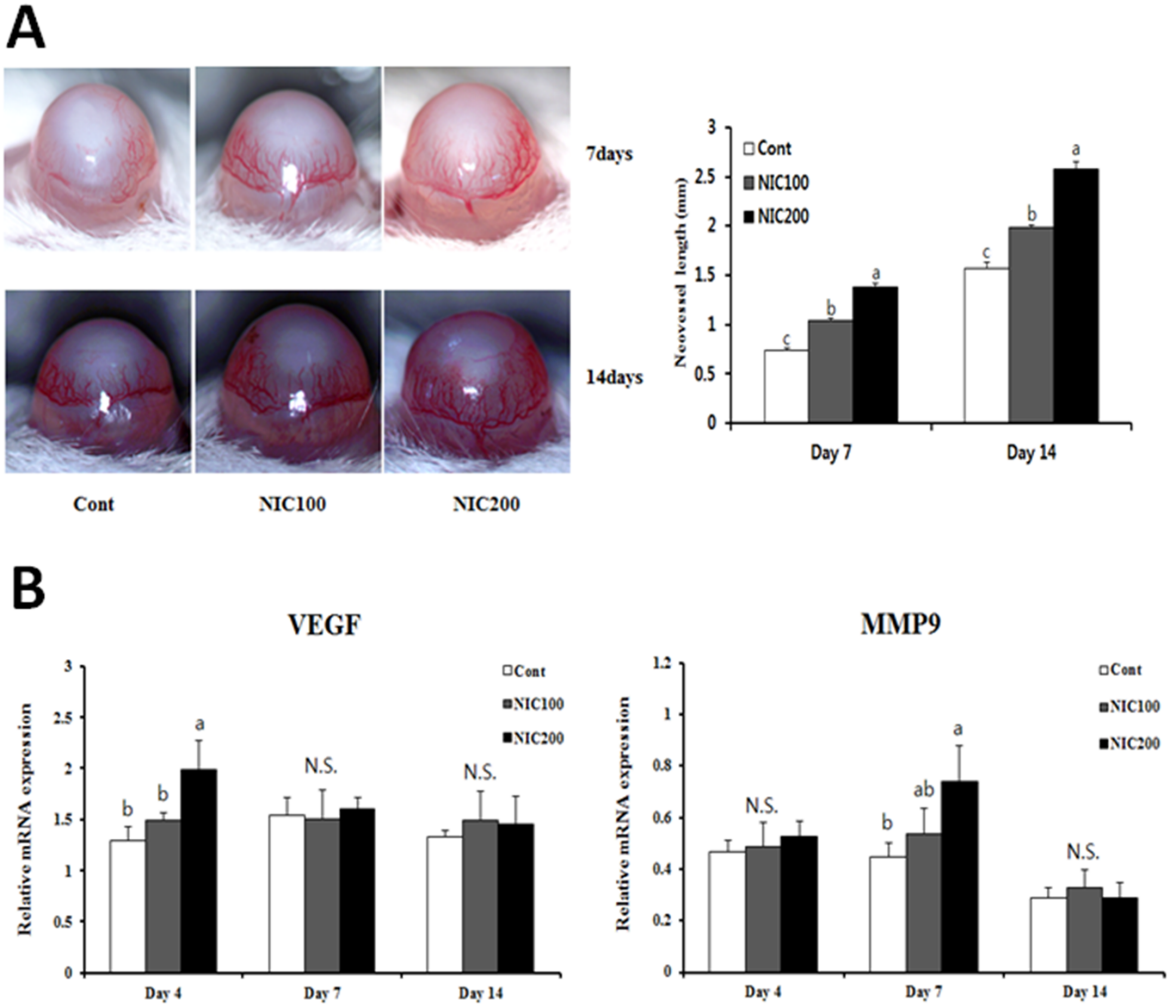
Alkali injury-induced corneal NV in corneas with or without NIC treatment. (A) Stereomicroscopic appearance of mouse eyes on days 7 and 14. The length of new blood vessels was determined. (B) Results of qRT-PCR to detect VEGF and MMP9 mRNA levels are shown. Data are expressed as the mean ± SEM per group and analyzed by ANOVA. Experimental groups marked by different letters (a, b, or c) represent significant difference at p<0.05 compared to the control group on each day. N.S., not significant.

To assess the effects of nicotine on the angiogenic response in corneal tissue, we first examined NV in mouse corneas after alkali injury. Since new blood vessels were observed within the first week following corneal alkali burn [29], we confirmed the presence of those new vessels in corneal tissue on days 7 and 14 post-injury. In line with the previous report, stereomicroscopic observation showed limbal vessels sprouting into the cornea on day 7 after the burn injury. On day 14 post-injury, a greater number of neovascular structures were observed in the cornea compared to those seen on day 7. Interestingly, increased vessel area and length were observed in the group treated with nicotine 7 or 14 days after injury. Also, such symptoms became more severe in the nicotine-treated group in a dose-dependent manner (Fig 1A). To further elucidate the effects of nicotine on the formation of new blood vessels, we next analyzed the mRNA expression of the angiogenesis-related genes vascular endothelial growth factor (VEGF) and MMP9 in corneal tissue following alkali injury. VEGF and MMP9 expression were significantly increased in mice treated with a high concentration of nicotine compared to control mice on days 4 and 7, respectively (Fig 1B). Overall, our results showed that nicotine administration enhances NV in injured corneas.

### Nicotine administration promotes fibrosis in corneal tissue after alkali burn injury

**Fig 2:**
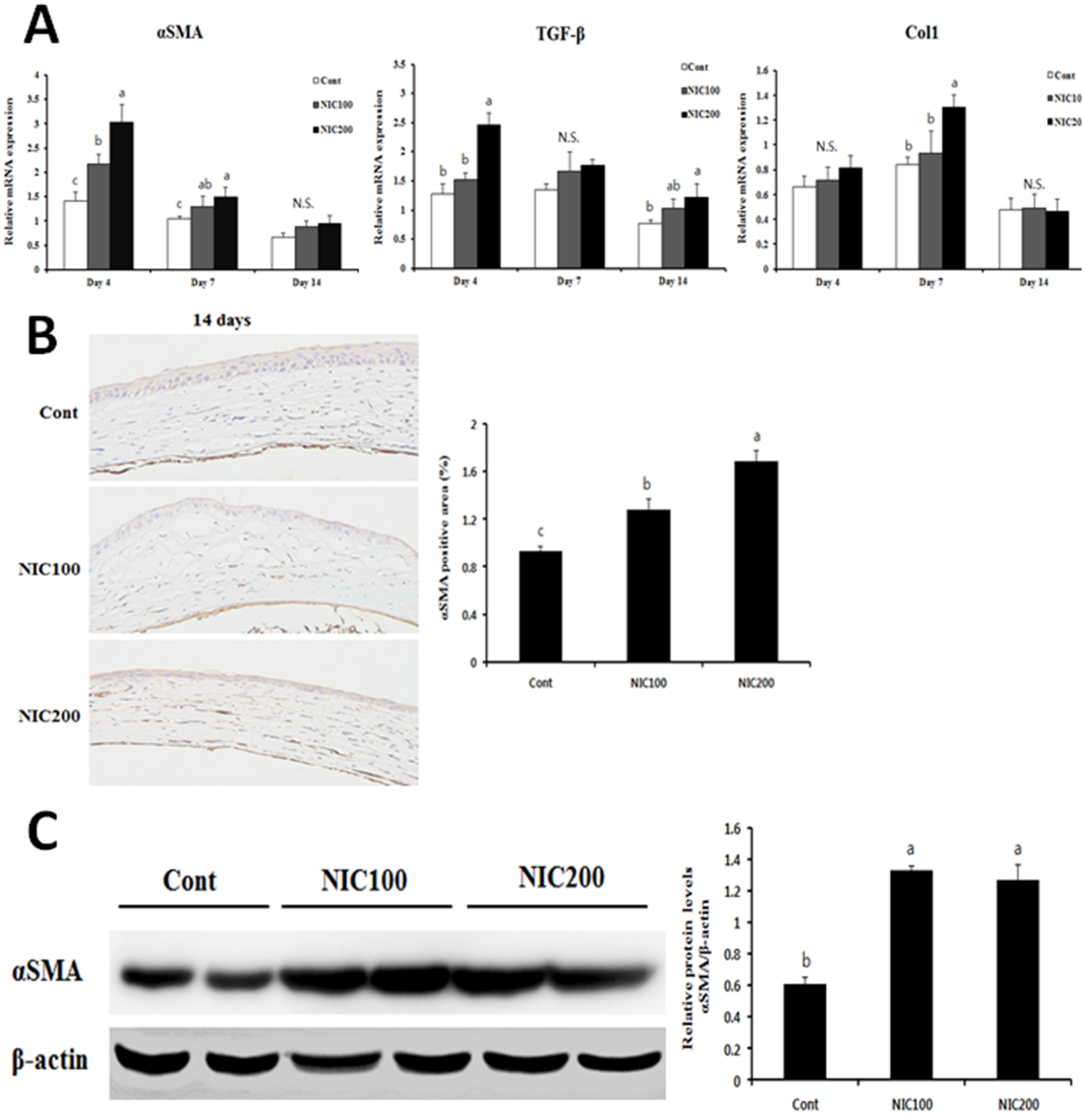
Evaluation of fibrogenic gene expression in injured corneal tissue. (A) mRNA levels of the fibrogenic genes αSMA, TGFβ1, and Col1 were analyzed. (B) Corneal tissues 14 days after injury were stained with an anti-αSMA antibody. Pictures are shown at 200x magnification. The percentage of αSMA-positive area was evaluated in corneal tissues. (C) αSMA protein levels were determined by Western blotting in corneal tissues with or without NIC treatment 14 days after alkali burn injury. Values are expressed as the mean ± SEM. Data was analyzed by ANOVA. Experimental groups marked by different letters (a, b, or c) represent significant difference at p<0.05 compared to the control group on each day. N.S., not significant.

Since alkali injury induces tissue fibrosis, resulting in scarring and opacification of the cornea, we evaluated the levels of the profibrotic genes αSMA, TGF-β, and Col1 at the indicated time points after injury. As shown in Fig 2A, the expression of fibrosis-related genes was markedly up-regulated by nicotine administration in 200 μg/ml nicotine-treated mice compared to control mice. αSMA mRNA expression was also significantly increased in 100 μg/ml nicotine-treated mice compared to control mice. It is well documented that keratocyte differentiation resulting in formation of myofibroblasts that express αSMA is one of the hallmarks of the corneal wound healing response. Thus, we next performed immunostaining to detect αSMA in corneal tissue after injury with or without nicotine treatment. The data confirmed that the αSMA-positive area was significantly increased by nicotine treatment in a dose-dependent manner on day 14 post-burn (Fig 2B). Finally, we further evaluated the levels of αSMA by using Western blot analysis to verify the immunostaining observation. The level of αSMA protein in corneal tissue was markedly augmented in nicotine-treated mice compared to control mice, but there was no significant difference between the nicotine-treated groups. These results suggest that nicotine treatment might affect the differentiation of fibroblasts to myofibroblasts and enhance the expression of αSMA in corneal tissue following alkali injury.

Collectively, these data indicate that nicotine administration could promote fibrotic tissue repair associated with corneal opacification and vision loss after chemical injury.

### The expression of nAChRs in corneal fibroblasts and tissues is increased by alkali burn injury

**Fig 3:**
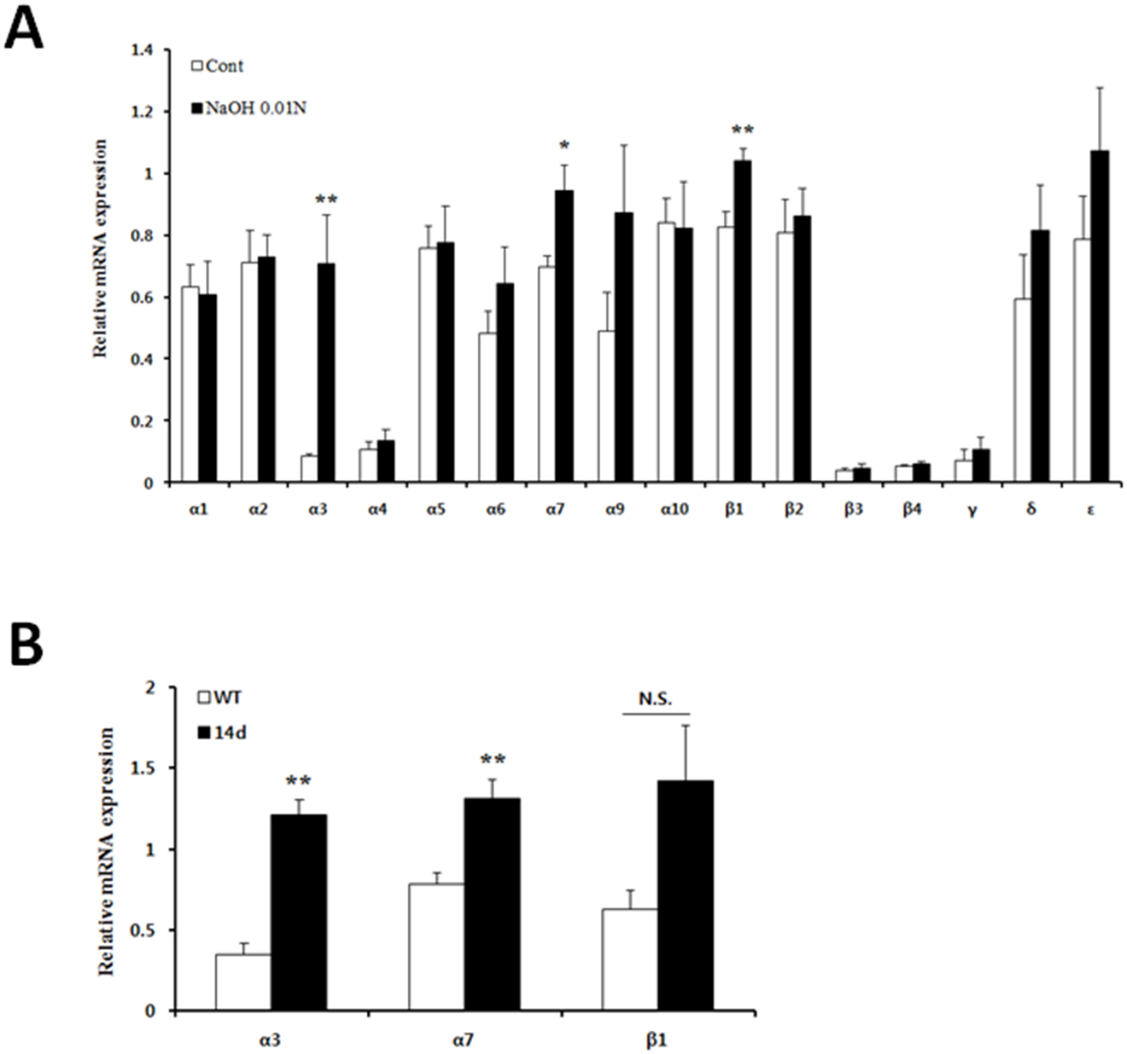
Comparison of nAChR subunit expression. (A) A representative qRT-PCR analysis showing expression of the α1–10, β1–4, γ, δ, and ε nAChR subunits in corneal fibroblasts. Values presented are from three independent experiments and each sample was assayed in duplicate. (B) mRNA levels of α3, α7, and β1 nAChR subunits in corneal tissues 14 days after injury. Data are expressed as the mean ± SEM per group and analyzed by Two-tailed Student’s *t* test; *P < 0.05, **P < 0.01. N.S., not significant.

Since nicotine exerts harmful effects on corneal wound healing, we investigated whether chemical injury affects the expression of nicotinic receptors. Among nAChR subtype genes, the mRNA levels of the α3, 7, and β1 nAChR subunits were significantly increased in 0.01N NaOH-treated corneal fibroblasts compared to untreated cells (Fig 3A). Thus, we next evaluated the expression of these genes in mice corneal tissues on day 14 following injury. The results showed that the levels of the α3 and 7 nAChR subunits were markedly augmented in alkali-injured corneal tissues compared to non-injured corneal tissues. Also, the expression level of the β1 nAChR subunit tended to increase, but there was no significant difference between the groups (Fig 3B).

Taken together, the increases in these nAChR subunits induced by chemical injury might have a negative influence on the alkali burned corneal wound healing response in mice treated with nicotine.

### Nicotine and cotinine treatment induce the expression of fibrotic genes in alkali-treated corneal fibroblasts

**Fig 4:**
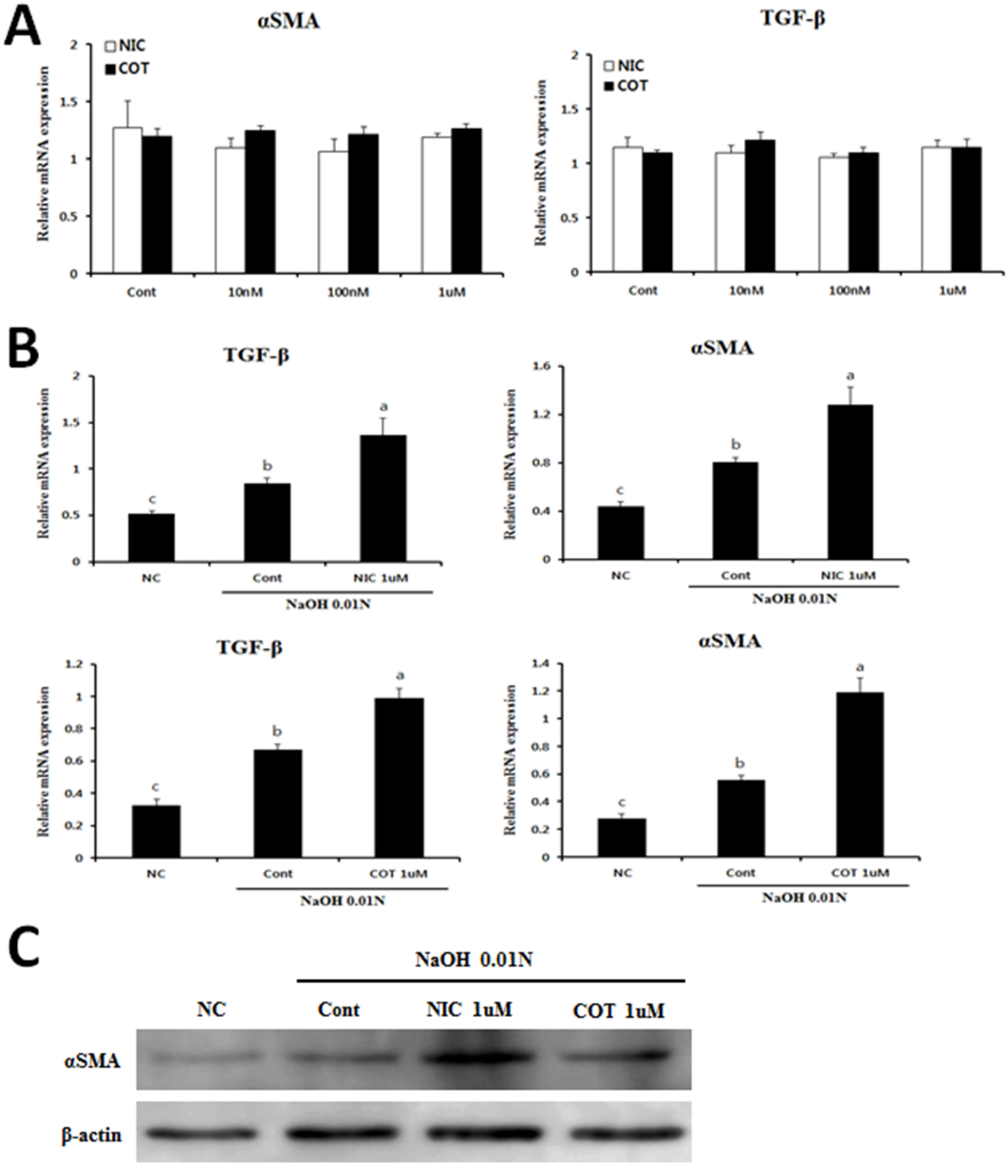
Assessment of the levels of fibrogenic genes in human corneal fibroblasts. (A) Cells were treated with the indicated concentration of NIC or COT. After 24 hours’ incubation, mRNA expression of αSMA and TGFβ1 was determined by qRT-PCR (B) Cells were treated with the indicated concentrations of NIC or COT along with 0.01N NaOH to induce a chemical injury. After 24 hours’ incubation, mRNA expression of αSMA and TGFβ1 was determined by qRT-PCR. (C) Protein levels of αSMA were determined by Western blotting in total cell lysates. Values presented are from two independent experiments and each sample was assayed in duplicate. Data are expressed as the mean ± SEM per group and analyzed by ANOVA. Experimental groups marked by different letters (a, b, or c) represent significant difference between groups at p<0.05.

To further investigate the role of nicotine on the fibrotic response in corneal fibroblasts, we assessed the expression of the fibrotic genes αSMA and TGF-β in corneal fibroblasts for 24 hours with or without nicotine or cotinine, which is one of the major metabolites of nicotine. Unexpectedly, the results showed that the indicated concentrations of nicotine and its metabolite did not affect the expression of fibrosis-related genes in corneal fibroblasts (Fig 4A). Thus, we next evaluated whether fibrotic gene expression was affected by treatment with the two compounds in alkali-injured corneal fibroblasts. Interestingly, the expression levels of these genes were significantly augmented by 0.01N NaOH treatment. Moreover, this effect was further elevated by treatment with nicotine or cotinine at the indicated concentrations (Fig 4B). Furthermore, similar patterns were observed in the levels of αSMA protein, as confirmed by Western blotting (Fig 4C).

Our results revealed that nicotine or cotinine combined with alkali injury synergistically exert fibrotic activity in corneal fibroblasts.

### Proliferation of corneal fibroblasts induced by nicotine and cotinine treatment is decreased by inhibition of the PI3K and PKC signaling pathways

**Fig 5:**
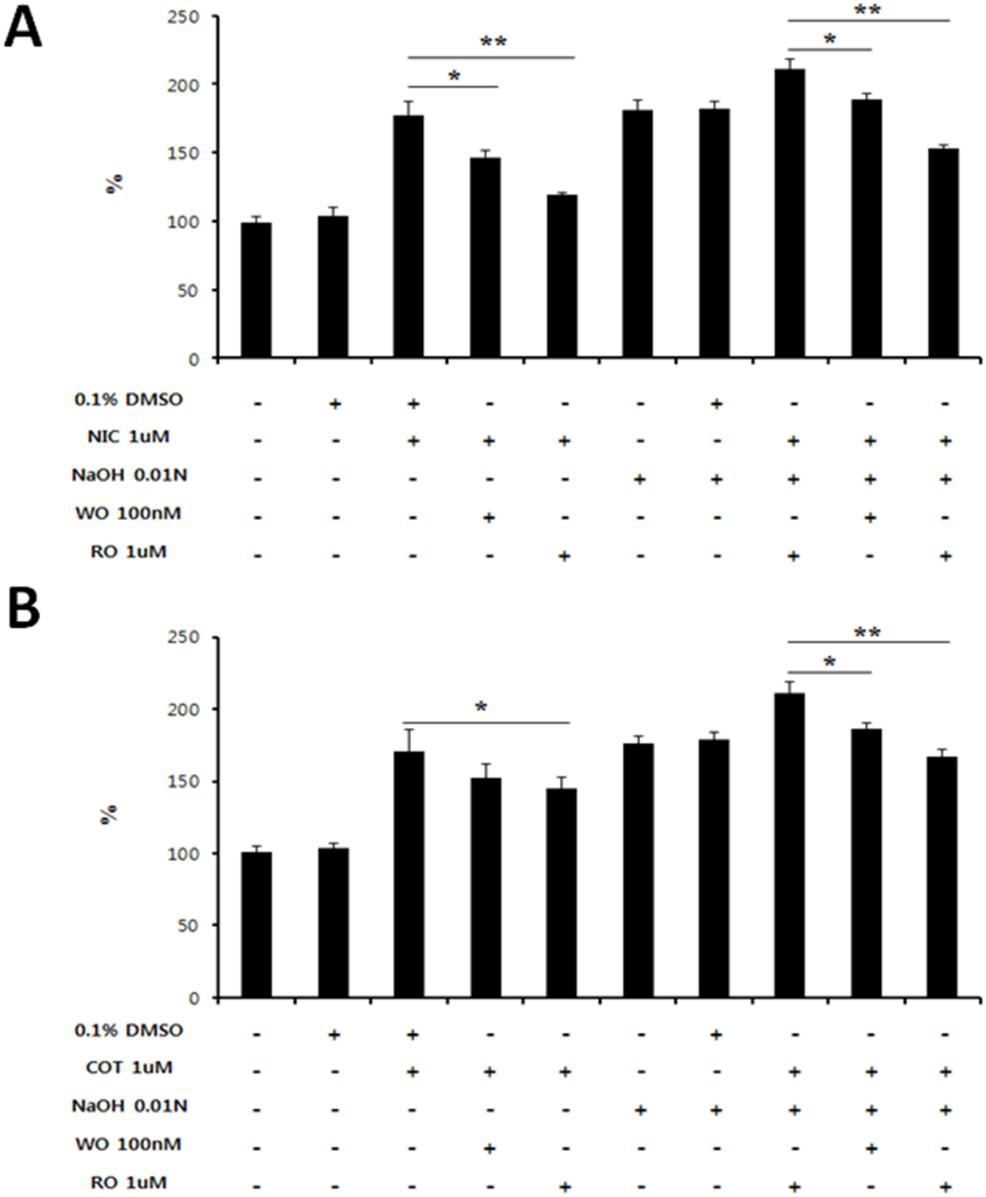
Determination of the intracellular signaling molecules involved in corneal fibroblast proliferation induced by treatment with NIC or COT. The specific inhibitors WO and RO were used to inhibit PI3K and PKC, respectively. (A and B) Cell proliferation was determined by the CCK-8 assay. Values presented are from three independent experiments. Data are expressed as the mean ± SEM per group and analyzed by Two-tailed Student’s *t* test; *P < 0.05, **P < 0.01.

To evaluate if the fibrotic effects of nicotine or cotinine are mediated by proliferation of corneal fibroblasts, cells were incubated with nicotine or cotinine plus specific inhibitors, WO or RO. It is well documented that nicotine is involved in several intracellular signaling pathways, such as the janus-activated kinase/STAT, PKC, PI3K/Akt, and Ras/Raf/MEK/ERK pathways [30, 31]. One recent study indicated that nicotine could induce hepatic stellate cell proliferation mediated by PI3K and PKC and contribute to hepatic fibrosis in chronic liver disease [7]. Also, nicotine accelerates the progression of chronic kidney disease by increasing mesangial cell proliferation via PKC activation [32]. Since nicotine and cotinine treatment increase the transdifferentiation of corneal fibroblasts following alkali injury because of an increase in αSMA expression (Fig 4B and 4C), we assessed whether these signaling pathways are involved in proliferation of these cells induced by treatment with nicotine or cotinine with or without 0.01N NaOH. We observed a marked increase in proliferation of these cells following treatment with 1 μM nicotine or cotinine as well as 0.01N NaOH treatment (Fig 5A). When nicotine or cotinine were administered together with 0.01N NaOH, proliferation of these cells was markedly increased. Interestingly, such effects were significantly reduced by treatment with WO and RO, which block the PI3K and PKC signaling pathways, respectively (Fig 5A and 5B).

These data clearly demonstrated that nicotine and cotinine promote proliferation of corneal fibroblasts through PI3K and PKC activation.

## Discussion

Previous studies focusing on the effects of nicotine on histological changes in eyes indicated that nicotine exposure induces a reduction in total retinal thickness in normal and diabetic mice [33]. Also, nicotine caused a significant decrease in choroidal thickness following oral administration by reduction of ocular blood flow [34]. Furthermore, subcutaneous injection of nicotine was found to be related to some morphological changes in the retina and lens, such as a decrement in retinal ganglion cell density, atrophy of the retinal nerve fiber layer, and thinning of the lens capsule [35]. Nicotine also affects the morphology and function of retinal pigment epithelial cells [36], indicating that the effects of nicotine may be involved in smoking-related retinal diseases. Several studies have provided clear evidence that nicotine promotes choroidal neovascularization because its mechanism of action involves an increase in proangiogenic VEGF and a decrease in antiangiogenic pigment epithelium-derived factor in retinal pigment epithelial cells [37], and nicotine facilitates recruitment of bone-marrow-derived cells into choroidal neovascularization lesions [38]. Thus, topical administration of mecamylamine, which is nonselective nAChR antagonist, could ameliorate choroidal NV associated with AMD in the presence or absence of nicotine stimulation [39].

In the present study, we found that chronic nicotine administration for 3 weeks did not affect corneal morphology in mice (data not shown). It is conceivable that the metabolites of nicotine may not affect the normal condition of corneal tissue because of its avascularity. Similar to our current findings, a recent clinical study indicated that no significant difference was observed in mean corneal endothelial density, central corneal thickness, or endothelial morphology between smokers and non-smokers [40]. However, alkali burned corneal tissue was affected by nicotine treatment, suggesting that newly formed blood vessels may be involved in the pathological actions of nicotine. Once corneal NV develops following a traumatic injury, it is further accelerated by nicotine and/or its metabolites, including cotinine. Our results also indicated that chronic nicotine administration could promote corneal NV on days 7 and 14 after alkali burn. Moreover, we observed increased expression of the proangiogenic genes VEGF and MMP9 in injured corneas following nicotine administration.

Since traumatic corneal injury induces the corneal fibrogenic response, we also assessed the effect of nicotine on the corneal fibrosis-related wound healing response. We found that chronic nicotine administration accelerated fibrogenesis in injured corneas. This effect appeared to be a consequence of increased activation of keratocytes because an increased number of αSMA-positive cells were observed in the corneal stroma of nicotine-treated mice after alkali injury. In accordance with this result, the mRNA and protein levels of αSMA were markedly increased in alkali injured corneal fibroblasts following treatment of nicotine or cotinine. Recent studies supporting our findings showed that nicotine modulates differentiation of lung fibroblasts and hepatic stellate cells, resulting in increased expression of αSMA [7, 41]. Moreover, we observed that alkali injury increased the mRNA levels of α3, 7, and β1 nAChRs in corneal fibroblasts and corneal tissues on day 14 following injury, except β1 nAChR. Although we did not confirm the protein levels of nAChR subunits, increased expression of these receptors induced by alkali injury might amplify the action of nicotine on injured corneal tissues as well as corneal fibroblasts.

Several recent studies indicated that nicotine significantly up-regulated the expression of TGF-β1 and its receptor and stimulated the expression of fibrosis-related genes [7, 42, 43]. Similar to these observations, our results showed that the mRNA levels of TGF-β1 were also augmented in alkali-treated corneal fibroblasts with nicotine or cotinine treatment. It is well known that TGF-β is a critical regulator involved in wound healing-related fibrosis, repair and differentiation in many tissues and cell types. Previous studies have suggested that TGF-β plays a pivotal role in the transformation of keratocytes to myofibroblasts, ECM production, and consequent corneal fibrogenesis [23, 44]. Thus, nicotine and increased TGF-β1 may act synergistically to induce the fibrogenic response in corneal fibroblasts and corneal tissues following chemical injury.

When corneal tissue is injured, corneal keratocytes undergo apoptotic cell death adjacent to the injury site, resulting in activation and proliferation of the remaining keratocytes. They then migrate toward the damaged site, and subsequently take on a repair phenotype similar to that of typical fibroblasts. As wound healing progresses, fibroblasts can transform into αSMA-positive myofibroblasts, resulting in deposition of ECM in injured corneal tissue. However, excessive activation of keratocytes and ECM deposition in injured cornea contribute to corneal opacity that results in visual impairment [45–47]. We herein demonstrated that proliferation of corneal fibroblasts was significantly increased by treatment with nicotine or cotinine. These findings are consistent with other reports regarding the effect of nicotine on the proliferation of other cell types, such as hepatic stellate cells [7] and human umbilical vein endothelial cells [48] at the same concentration used in our *in vitro* experiments. The proliferation of these cells was augmented by alkali injury, and the effect was further increased by treatment with 0.01N NaOH along with nicotine or cotinine.

A number of signaling cascades affected by nicotine have been identified in non-neuronal cells [30]. Although we did not confirm all signaling pathways related to nicotine and/or cotinine, the results of the current study indicated that the mechanism of action by which nicotine causes proliferation of corneal fibroblasts is associated with the PKC and PI3K signaling pathways. Similar to our results, a recent study indicated that TGF-β1-induced corneal fibroblast transdifferentiation was significantly inhibited by a specific inhibitor of the Akt singlaing pathway, indicating that PI3K/Akt signaling is downstream of Smad-independent TGF-β signaling [49]. Another interesting recent finding further supports our data: chondrocyte-derived ECM was shown to suppress alkali burn-induced corneal NV and fibrosis by inhibition of NF-κB activation through blockade of the Akt and PKC signaling pathways [50].

Ocular trauma resulting from a chemical burn manifests as complicated clinical symptoms, such as corneal epithelial defects, scar formation, angiogenesis, tissue inflammation, and fibrosis, leading to reduced corneal transparency and vision loss [51]. Among these symptoms, corneal reepithelialization is pivotal for successful healing as it decreases the risk of infection associated with increased inflammatory and fibrogenic responses [52]. A recent study indicated that increased PI3K/Akt signaling was involved in a significant enhancement of human corneal epithelial cell migration and cornea scratch wound healing [53]. Also, exogenously added TGF-β3 accelerated corneal epithelial wound healing via Smad and PI3K/Akt signaling in diabetic mice. Furthermore, PKC-alpha activity increased during corneal epithelial wound healing, which stimulated hepatocyte growth factor-induced corneal epithelial cell proliferation and migration [54, 55]. Therefore, it is valuable to verify the exact effects and outcomes of inhibiting PKC and PI3K/Akt signaling in chemically burned cornea associated with epithelial wound healing, angiogenesis, and fibrosis. Additionally, although a similar transduction pathway was observed regarding TGF-β-induced myofibroblast differentiation in a cultured human corneal fibroblast cell line compared to normal cultured human corneal keratocytes [56], it is necessary to assess the effect of nicotine or cotinine and associated signaling pathways in human primary corneal keratocytes.

In summary, our results provide clear evidence that chronic nicotine adiminstration accelerates corneal angiognesis and fibrosis following alkali injury. The effects of nicotine were mediated, at least in part, by PI3K and PKC signaling.
